# Independent medical evaluation for sick-listed patients: a focus group study of GPs´ expectations and experiences

**DOI:** 10.1186/s12913-018-3481-3

**Published:** 2018-08-29

**Authors:** Aase Aamland, Elisabeth Husabo, Silje Maeland

**Affiliations:** 1 Research Unit for General Practice, Uni Research Health, Bergen, Norway; 2 Uni Research Health, Uni Research, Bergen, Norway

**Keywords:** General practice, Sick-leave, Independent medical evaluation, Focus groups, Qualitative research

## Abstract

**Background:**

Norwegian general practitioners (GPs) are important stakeholders because they manage 80% of people on long-term sick-leave. Independent medical evaluation (IME) for long-term sick-listed patients is being evaluated in a large randomized controlled trial in one county in Norway in an effort to lower the national sick-leave rate (the NIME trial: Effect Evaluation of IME in Norway). The aim of the current study was to explore GPs’ expectations of and experiences with IMEs.

**Methods:**

We conducted three focus group interviews with a convenience sample of 14 GPs who had had 2–9 (mean 5) of their long-term sick-listed patients summoned to an IME. We asked them to recollect and describe their concrete expectations of and experiences with patients assigned to an IME. Systematic text condensation, a method for thematic cross-case analysis, was applied for analysis.

**Results:**

To care for and to reassure their assigned sick-listed patients, the participants had spent time and applied different strategies before their patients had attended an IME. The participants welcomed a second opinion from an experienced GP colleague as a way of obtaining constructive advice for further sick-leave measures and/or medical advice. However, they mainly described the IME reports in negative terms, as these were either too categorical or provided unusable advice for further follow-up of their sick-listed patients. The participants did not agree with the proposed routine use of IMEs but instead suggested that GPs should be able to select particularly challenging sick-listed patients for an IME, which should be performed by a peer.

**Conclusion:**

Our participants showed positive attitudes towards second opinions but found the regular IMEs to be unsuitable. The participants did however welcome IMEs if they themselves could select particularly challenging patients for a mandatory second opinion by a peer but emphasized that IME-doctors should not be able to overrule a GP’s sick-leave recommendation. These findings, together with other evaluations, will serve as a basis for the Norwegian government’s decision on whether or not to implement IMEs for long-term sick-listed patients.

**Trial registration:**

ClinicalTrials.gov NCT02524392. Registered 23 June, 2015.

**Electronic supplementary material:**

The online version of this article (10.1186/s12913-018-3481-3) contains supplementary material, which is available to authorized users.

## Background

The Norwegian government is seeking new strategies to lower the country’s sick-leave rate, which currently is at 6.4% [1] and claimed to be among Europe’s highest [2]. Norwegian general practitioners (GPs) are important stakeholders because they manage 80% of people on long-term sick-leave [3]. Two reviews show how GPs worldwide report conflict with the multiple roles they have to fulfil when certifying sickness absences, and the two reviews indicate that GPs feel more competent in treating patients than acting as society’s gate-keepers [4, 5]. Furthermore, when promoting return to work (RTW) among their sick-listed patients, GPs describe challenges with assessing work ability, the percentage of functional work-capacity and the length of sick-leave periods [4, 5]. If the patient is reluctant to RTW, the GP may avoid the issue to preserve a good relationship [4, 5]. Denying a patient’s request for sick-leave might come at a high price, for instance the patient might change her GP, and thus terminate a close doctor-patient relationship which may have a strong emotional impact on GPs [6].

In an effort to lower the country’s sick-leave rate, the Norwegian Ministry of Labour and Social Affairs has proposed the implementation of independent medical evaluations (IMEs) for all patients who have been sick-listed for six months. IMEs conducted by independent health professionals are routinely used by both public and private health insurance systems throughout the Western world to determine the functional capacity of workers who claim inability to work because of illness or injury [7–9]. However, experts assessing the same claimants often disagree regarding the ability to work [10–12]. An IME implies that, by providing a second opinion, the IME doctor intervenes in a sickness absence episode and hence in the relationship between the sick-listed patient and her GP. Both the patients and the GPs are challenged as the underlying assumption from the insurers seems to be that the patients are fraudulent or malingering [13], and that GPs, to protect vulnerable patients, are colluding with the patients and are less cooperative with the insurer [14].

To evaluate whether IME has an effect on return to work (RTW) after long-term sick-leave, a large randomized controlled trial in one representative Norwegian county was designed and initiated before a possible national implementation. The NIME trial (Effect Evaluation of IME in Norway) [15] is the first controlled evaluation of IMEs worldwide. In this trial, the IME doctors are specially trained GPs employed by the Norwegian Labour and Welfare Administration. A summary from the treating GP is provided to the IME doctor in preparation for the consultation. The main purpose of the IMEs is to contribute with new perspectives on the sickness absence episode, to assess work ability, to explore the patient’s own expectations and perceived barriers for RTW, and to make suggestions for further follow-up measures and the level of sick leave, in a written report to the treating GP. The IME doctors cannot overrule the treating GP’s assessments of sick-leave [15]. The NIME trial will be evaluated through assessing the effects on RTW, cost–benefits analysis, and qualitative interviews with the three key stakeholders. In two earlier studies, we have explored expectations of and experiences with IMEs from the sick-listed patients´ [16] and the IME-doctors´ [17] points of view. In this current study, we explore GPs’ expectations of and experiences with IMEs, thus completing the triangulation. No other studies have evaluated GPs´ perspectives on IMEs. The results from the entire evaluation will be of great importance when assessing the usability of IMEs as a means to lower the Norwegian sick-leave rate.

## Methods

### Recruitment and sample

We intended to select a purposive sample of GPs in terms of gender, work experience and a minimum number of three patients enrolled in the NIME trial. The project secretary invited 20 GPs, but only one GP responded to our initial and reminder invitation letter. Based on previous experience, it is challenging to recruite Norwegian GPs for research purposes, so we decided to change our recruitment strategy. The first author (AA) personally telephoned and/or emailed potential participants on a list provided by the project secretary. AA offered the potential participants information about the purpose of the study and suggested they would be remunerated for their participation. Data were drawn from three focus groups with a total of 14 participants. Our convenience sample of GPs included nine male and five female GPs, aged 32–64 (mean 49) years. Three of the GPs were in the process of completing their formal specialization, whereas the others had been GP specialists for many years. They had worked as GPs for 3–33 years (mean 17) and had had 2–9 (mean 5) of their sick-listed patients enrolled in the NIME trial.

### Data collection

The focus group interviews lasted about 90 min and took place in three different general practices belonging to one of the participating GPs in each focus group. The focus group interviews were conducted by AA using a semi-structured interview guide (Additional file 1). To stimulate a focused discussion, we started the interviews by asking each participant to describe one concrete IME-case. The participants thereafter actively shared and reflected on their similar or different expectations and experiences. EH was an observer and took notes. At the end of the interviews, she summarized her impression of the comments, and sought clarity in order to correct potential misunderstandings. The interviews were audiotaped and transcribed verbatim.

### Analysis and information power

We used systematic text condensation for analysis [18]. This is a thematic cross-case analysis that comprises four steps: (i) reading all the material to obtain an overall impression and to recognize preliminary themes; (ii) developing code groups from the preliminary themes by identifying meaning units that reflect different aspects of the participants’ expectations and experiences, and then coding for these; (iii) identifying subgroups within each code group that exemplify the vital aspects of each code group by condensing the contents of each of these and identifying illustrative quotations for each subgroup; and (iv) synthesizing the condensates from each code group and presenting a reconceptualized description of each category to include the different accounts of expectations and experiences of IMEs among our participants. All three authors performed the analysis together.

We used Lipsky’s theory of street-level bureaucracy [19] to plan the study and discuss our findings, as it describes and explains the complex dynamics between state, bureaucrats and citizens. Lipsky describes street-level bureaucrats as the face of policy since these individuals interact directly with citizens. Based on the theory, GPs act as gate-keepers when assessing their patients’ entitlements to sick-leave and may be concerned with how they should uphold policy.

An assessment of information power [20] guided our sample size by considering the following aspects: the study has a narrow aim, thus focusing the study, and we used an established theory (Lipsky). Furthermore, the quality of dialogue was good in all three interviews, and intensive in two of them. The participants shared expectations and experiences with each other and built upon each other’s comments. Finally, our analysis strategy was thematic cross-case, and followed the set strategy elaborated above. All this considered, we judged the information power to be sufficient for performing the analysis after three interviews.

## Results

Our analysis revealed a broad range of GPs’ expectations and experiences of IMEs, which are elaborated below. The participants who provided the quotations have been assigned pseudonyms.

### GPs had positive attitudes towards being checked by another GP but taking care of patients assigned to an IME could be time-consuming

Many participants mentioned that although their spontaneous reaction to having their work checked by another doctor had evoked feelings of discomfort, these feelings soon turned into acceptance as they became aware of their role as a gate-keepers to the society’s resources. They reflected on the possibility of having overlooked medical cues or possible sick-leave measures for their patients. One participant described how she trusted the IME doctors because she considered them to be highly experienced colleagues with strong professional skills. Most of the participants noted the importance of having their work checked by another experienced GP. However, the participants’ reactions were mainly negative when they became aware that some IME doctors in other countries are not GPs but have other specializations such as orthopedic surgeons. They used words such as ‘hopeless’, ‘confusing’ and ‘counter-productive’ to describe their attitudes to non-GPs acting as IME doctors.

A male participant in his 60s who had worked as a GP for 30 years welcomed second opinions:

*“I think it’s okay that others judge my work as sufficient or not. I believe it will become more and more common in the future… to be checked… whether our work maintains a desired quality or not, both in regards to sick-listing and other parts of our work.”* (Bill).

The participants described the different reactions of the patients who had been summoned to an IME consultation. Some patients contacted their GP because they were apprehensive and did not understand the purpose of the IME consultation, whereas others booked an extra consultation with their GP to obtain more information. Some participants had not talked to their patients before an IME, whereas a few GPs got in touch with their patients directly to provide them with information and support. Most participants described a number of different strategies to reduce their patients’ feelings of stress and anxiety about the IME. First, some participants would emphasize to their patients that the IME was an ongoing project and that the patients had been randomly assigned to the IME consultation. Second, to reassure their patients, a few participants would note that the IME doctor could not overrule the GP’s assessment of ongoing sick-leave. Third, many participants would point out that the IME was more of a means of monitoring GPs than of checking patients when trying to determine whether patients were legitimately sick-listed. A female participant in her 60s described how she had systematically encouraged her patients to attend the IME consultations by emphasizing this point:

*“I think my patients regarded us as allies against those who would scrutinize my work.”* (Kate).

### GPs with an in-depth knowledge of patients should be able to select challenging patients to attend a mandatory IME and to spare already clarified patients

Several participants viewed knowing their patients’ life experiences as a strength of general practice. They found it challenging having to retell the background and reasons for ongoing sick-leave in a short summary and questioned whether an IME doctor could grasp the whole picture based on one short encounter. On the other hand, some participants noted that long-standing patient relationships could also produce locked patterns and/or blind spots, and they therefore welcomed a second opinion from a colleague. A male participant who had known some of his sick-listed patients from their childhood commented on the importance of a long relationship like this:

“*I know so many things that happened to that patient a very, very long time ago that are likely to shape who he is today. And you can’t express all that in a summary….”* (Luke).

Many participants had experienced inexpedient inclusions of sick-listed patients in an IME. They described such patients as medically clarified with already well-defined plans for RTW. One example was a patient with a bipolar illness for whom further treatment and a detailed plan for RTW had already been drawn up. Several participants stated how they would prefer to select challenging patients themselves when they felt a need for advice; for instance, in situations where the GP and the patient did not share a common view on the patient’s work ability. Some participants felt frustrated if these patients had been randomized to an IME and had chosen not to attend. A male participant described a patient who worked in a grocery store and experienced diffuse shoulder pain, and who still felt unable to work even after an operation and long-term physiotherapy. This participant had been looking forward to his patient attending the IME and was disappointed when the patient had chosen not to attend. Another participant described a patient with long-standing diffuse abdominal pain who, after the IME consultation, seemed to have gained an enhanced acceptance of the need to cope with the distress instead of continuing to search for a cure. A male participant in his 50s who had worked as a GP for 20 years voiced a wish to actively select the challenging sick-listed patients who were to attend a mandatory IME:

*“It is much more goal-oriented if the sick-listing doctor selects difficult patients.”* (Paul).

### No or perceived useless suggestions in the IME reports evoked negative attitudes to the intervention and led GPs to request enhanced use of already existing interventions

Most participants felt that the IME reports did not contain any useful advice for further follow-up. Some participants found that the reports from IME doctors were too categorical, and this had evoked feelings of surprise and amusement as well as disappointment and frustration. A few participants wondered whether the IME doctor sometimes felt the need to make suggestions to justify their position. A female participant described her disappointment with one such report after having spent much time writing the summary and hoping to receive collegial advice.

Many IME doctors had suggested referral to a psychologist. However, many of the participants felt these were unhelpful suggestions for several reasons: patients had tried psychological counselling before or did not want to try it, the timing was not right, or the psychology services had long wait-lists and limited availability. Some participants noted that many GPs also provide systematic cognitive therapy themselves as part of their regular interactions with patients.

One participant mentioned a counter-productive incident where the IME doctor had given the patient advice about a disability pension. The participant described how he had seen good potential for RTW; however, after the IME, the patient only wanted a disability pension. By contrast, several participants felt reassured when the IME doctor confirmed their work as appropriate and sometimes even gave explicit positive feedback. One participant was relieved when one of her patients described how the IME doctor had stressed that she was in good hands with her GP.

By contrast, another female participant in her 30s shared her disappointment about the lack of constructive feedback in the reports from the IME doctors:

*“The conclusions in all these reports are exactly the same as my conclusions… no new suggestions… there just wasn’t anything, just the status quo, and we were to continue.”* (Jane).

Several participants considered the IME to be another pointless intervention from the state and instead called for an enhanced use of already existing interventions. Examples included that the dialogue meeting should occur earlier than the usual six months of sick-leave, scheduling a billable second opinion by another GP, or discussing challenging cases in their continuing medical education groups. Although many of the participants welcomed receiving more knowledge about possible sick-leave measures embodied in the Norwegian Labour and Welfare Administration system, they felt that it would be more appropriate for case managers to proactively propose these. A male participant in his 50s who had worked as a GP for 22 years, summed this up as follows:

*“But… do we have to… I mean, we write our reports to Norwegian Labour and Welfare Administration… isn’t that enough for the case managers to make use of their interventions?”* (Sam).

## Discussion

Although it meant extra work, our participants had positive attitudes towards second opinions, but in the end, although they did not find the IME-reports to be useful. The participants suggested that GPs should be able to select particularly challenging sick-listed patients for a mandatory IME performed by a peer.

### Discussion of results and comparison with existing literature

The NIME trial forced extra work on the GPs through reassuring assigned patients and writing of resumes. A recent study exploring the sick-listed patients’ perspectives, reports the importance of GPs spending time and effort on these tasks [16]. Our participants had positive attitudes towards receiving second opinions as they acknowledged possible medical blind spots or lack of insight into useful sick-leave measures. This is consistent with previous research showing that GPs lack insight into important resources and/or measures in the RTW processes [21, 22], and is also in line with Lipsky’s theory that street-level-bureaucrats may lack knowledge in order to respond properly to individual cases [19]. Nevertheless, one of our main findings showed that the participants did not find the IME-reports useful, which is contrasting the IME-doctors own view of contributing with constructive second opinions [17]. For example, the participants expressed skepticism towards suggestions of referrals to a psychologist and noted a number of plausible reasons for this skepticism. This response may also be an example of lack of knowledge due to lack of time resources [19] to stay updated, particularly since the positive effect of cognitive behavioral interventions on RTW is well documented among patients with common mental disorders [23] and for patients experiencing long-term and functionally disabling unexplained symptoms [24]. On the other hand, receiving advice on a patient that they did not find challenging may explain the low perceived utility of the IME report. It appeared that our participants would have higher trust in IMEs if they could select particularly challenging sick-listed patients for a mandatory IME. In a Norwegian study aiming to lower sick-leave rate, the GPs picked complex sick-listed cases to discuss with senior insurance physicians. Yet, this study found that such supervision did not result in a decreased sick-leave rate, although the participating GPs expressed great satisfaction with the intervention [25]. Finally, our participants emphasized the importance of IME-doctors being GPs. Our participants trusted experienced peers (i.e. the IME doctors in the NIME trial) to be the most suitable doctors to perform IMEs. Findings from Australia support this as GPs [26] and psychologists [27] reported negatively to IMEs being performed by professionals who lacked experience and skills to evaluate the reasons for the patients’ sickness absence. Both our and the Australian findings may however raise an issue that is central in IME internationally: can doctors/health professionals accept a contrasting assessment by another health professional, essentially interfering in a long-established doctor-patient relationship? Previous research has shown that GPs struggle to accept it when the welfare administration overrules their sick-leave recommendations [28].

### Strengths and limitations

The participants shared their experiences in an atmosphere characterized by mutual trust, which was confirmed by our observations of spontaneous interactions between participants, follow-up questions and few interruptions or silent moments. This supported our choice of a focus group design because we wanted to take advantage of the communicative interactions between the participants as they shared their experiences [29]. However, to encourage a focused articulation of specific examples from their unique experiences with IMEs, the moderator had to intervene occasionally when the participants shared more general views about the Norwegian welfare system. This role of the moderator may have strengthened the internal validity of our study. All authors took part in the analysis, and we focused on distinguishing between what we thought we would find and what we actually found. By being a research team with different professional backgrounds (AA a GP, EH a psychologist, and SM a physiotherapist), our study may have been strengthed as this constellation of researches reduced the potential for professional blind spots. It was necessary to shift our strategy from an intended purposive sample to a convenience sample. This resulted in the inclusion of more male GPs than females and one GP who participated only had two patients in the NIME trial, but otherwise we retained diversity with regards to age and work experience.

## Conclusions

Our participants had positive attitudes towards second opinions but found the IMEs on a regular basis to be unsuitable. However, they welcomed mandatory IMEs, performed by peers, if they themselves could select particularly challenging sick-listed patients for an IME. It was emphasized that an IME should not overrule the treating GP’s sick-leave recommendations. These findings will, together with other findings from the evaluation of the NIME trial serve as a basis for the Norwegian government’s decision about the possible implementation of IMEs. Further, they can greatly inform implementation and management of IMEs in other contries as they present a group involved in IMEs not earlier studied.

## Additional file

Additional file 1: Focus Group Discussion Guide. (DOCX 19 kb)

## Abbreviations

GP: General practitioner
IME: Independent medical evaluation
NIME-trial: Effect Evaluation of Independent Medical Evaluation in Norway
RTW: Return to work

## References

[1] Sickness absence adjusted for seasonal and influenza variations (self- and doctor certified) in the 1st quarter of 2018: Statistics Norway; 2018 [updated 13 th June. Available from: https://www.ssb.no/en/arbeid-og-lonn/statistikker/sykefratot/kvartal.

[2] Organisation for Economic Co-operation and Development (2010). Sickness, Disability and Work: breaking the barriers. A synthesis of findings across OECD countries: OECD Publishing.

[3] Brage S, Bragstad T, Sorbo J (2014). Where are the sick- listed going?. Arbeid og velferd.

[4] Wynne-Jones G, Mallen CD, Main CJ, Dunn KM (2010). What do GPs feel about sickness certification? A systematic search and narrative review. Scand J Prim Health Care.

[5] Aamland A, Maeland S (2016). primary care physicians’ attitudes to and experiences with sick leave and follow-up. A scoping review. Tidsskr Velferdsforskn.

[6] Nilsen S, Malterud K (2017). What happens when the doctor denies a patient’s request? A qualitative interview study among general practitioners in Norway. Scand J Prim Health Care.

[7] Anner J, Kunz R, Boer W (2014). Reporting about disability evaluation in European countries. Disabil Rehabil.

[8] Anner J, Schwegler U, Kunz R, Trezzini B, de Boer W (2012). Evaluation of work disability and the international classification of functioning, disability and health: what to expect and what not. BMC Public Health.

[9] Busse JW, Bruun-Meyer SE, Ebrahim S, Kunz R (2014). A 45-year-old woman referred for an independent medical evaluation by her insurer. CMAJ.

[10] Ingravallo F, Vignatelli L, Brini M, Brugaletta C, Franceschini C, Lugaresi F (2008). Medico-legal assessment of disability in narcolepsy: an interobserver reliability study. J Sleep Res.

[11] Lax MB, Manetti FA, Klein RA (2004). Medical evaluation of work-related illness: evaluations by a treating occupational medicine specialist and by independent medical examiners compared. Int J Occup Environ Health.

[12] Elder AG, Symington IS, Symington EH (1994). Do occupational physicians agree about ill-health retiral? A study of simulated retirement assessments. Occup Med (Lond).

[13] Kilgour E, Kosny A, McKenzie D, Collie A (2015). Interactions between injured workers and insurers in workers' compensation systems: a systematic review of qualitative research literature. J Occup Rehabil.

[14] Lax M (2004). Independent of what? The independent medical examination business. New Solut.

[15] Husabo E, Monstad K, Holmås TH, Oyeflaten I, Werner EL, Maeland S (2017). Protocol for the effect evaluation of independent medical evaluation after six months sick leave: a randomized controlled trial of independent medical evaluation versus treatment as usual in Norway. BMC Public Health.

[16] Aamland A, Maeland S (2018). Sick-listed workers’ expectations about and experiences with independent medical evaluation: a qualitative interview study from Norway. Scand J Prim Health Care.

[17] Aamland A, Oyeflaten I, Maeland S. Independent medical evaluation for sick-listed workers in Norway: a focus group study of the experience of IME doctors. Scand J Public Health. 2017;1403494817745001. http://journals.sagepub.com.pva.uib.no/action/showCitFormats?href=%2Faction%2FdoSearch%3FAllField%3Daamland%26SeriesKey%3Dsjpc&mailPageTitle=Search+Results&doi=10.1177%2F1403494817745001. 10.1177/1403494817745001 29199916

[18] Malterud K (2012). Systematic text condensation – a strategy for qualitative analysis. Scand J Public Health.

[19] Lipsky M (2010). Dilemmas of the individual in public services. 30 Anv Exp edition. 2nd ed.

[20] Malterud K, Siersma VD, Guassora AD (2016). Sample size in qualitative interview studies: guided by information power. Qual Health Res.

[21] Letrilliart L, Barrau A (2012). Difficulties with the sickness certification process in general practice and possible solutions: a systematic review. Eur J Gen Pract.

[22] Mazza D, Brijnath B, Singh N, Kosny A, Ruseckaite R, Collie A (2015). General practitioners and sickness certification for injury in Australia. BMC Fam Pract.

[23] Reme SE, Grasdal AL, Lovvik C, Lie SA, Overland S (2015). Work-focused cognitive-behavioural therapy and individual job support to increase work participation in common mental disorders: a randomised controlled multicentre trial. Occup Environ Med.

[24] olde Hartman TC, Rosendal M, Aamland A, van der Horst HE, Rosmalen JG, Burton CD, et al. What do guidelines and systematic reviews tell us about the management of medically unexplained symptoms in primary care? BJGP Open. 2017; 10.3399/bjgpopen17X101061. 10.3399/bjgpopen17X101061 PMC6169926 30564678

[25] Mykletun A, Nilsen S, Kann IC, Jacobsen HR, Wesnes Ø, Nordhagen HP. NAV-doctorstudy in Bergen: Systematic supervison of GPs did not reduce the sick leave rate. Arbeid og velferd. 2015;3.

[26] Brijnath B, Mazza D, Singh N, Kosny A, Ruseckaite R, Collie A (2014). Mental health claims management and return to work: qualitative insights from Melbourne, Australia. J Occup Rehabil.

[27] Kilgour E, Kosny A, Akkermans A, Collie A (2015). Procedural justice and the use of independent medical evaluations in workers’ compensation. Psychol Inj Law.

[28] Arreloev B, Alexanderson K, Hagberg J, Loefgren A, Nilsson G, Ponzer S (2007). Dealing with sickness certification - a survey of problems and strategies among general practitioners and orthopaedic surgeons. BMC Public Health.

[29] Morgan D (1997). Focus groups as qualitative research.

