# Supplementary material for: Independent medical evaluation for sick-listed patients: a focus group study of GPs´ expectations and experiences
Source: BMC Health Serv Res. 2018 Aug 29;18:666. doi: 10.1186/s12913-018-3481-3 (PMC6114176; doi:10.1186/s12913-018-3481-3)
Supplement: Supplementary file 1 — Focus Group Discussion Guide. (DOCX 19 kb) [file 12913_2018_3481_MOESM1_ESM.docx]

### Focus group discussion guide: Independent medical evaluation for sick-listed patients: A focus group study of the expectations and experiences of GPs

### Introduction

Short presentation of the moderator. Our aim. Your role as participants. Practical information about: 1) recording & data storage, 2) your anonymity,3) ethical considerations regarding the importance of telling patient-trajectories in an anonymous way, 4) the moderator´s role (wanting free discussion, however, possibly need of clarifying questions and/or interruptions if you are not concrete enough or clearly leave the subject which we want you to discuss).

### Interview guide ( *a guide for structure more than a compulsive checklist during the discussion)*

**First question**

Can one of you start by describing one concrete experience which you had from being the GP to one patient who had been withdrawn for an IME? Please describe the whole trajectory from the day you got the call for a resume, your dialogue with the patient along the process, and finally how the IME report influenced your further follow-up of the actual sick-listed patient

**Knowledge of the NIME-trial**

- What did you know from before?

**Resume:**

- Considerations of the call for a resume
- Experiences from information and time-consume
- Considerations of not writing a resume (their reasoning for not doing it)
- Some positive “side-effects” from writing a resume for you as the GP?

**IME-report**

- Your expectations
- New insight into different sick-leave measures? If how, what?
- How did you use the reports in your further follow-up? If not, why not?
- Advices which you disagreed in? Why?

**Experiences from 2nd opinions from a collegue**

- Impressions of the IME doctors
- Loss of control?
- Way of getting help for further follow-up?
- Your dialogue and information to patients ahead

**Considerations of the timing of the IME consultations**
